# High-Throughput Screening of *Entamoeba* Identifies Compounds Which Target Both Life Cycle Stages and Which Are Effective Against Metronidazole Resistant Parasites

**DOI:** 10.3389/fcimb.2018.00276

**Published:** 2018-08-17

**Authors:** Gretchen M. Ehrenkaufer, Susmitha Suresh, David Solow-Cordero, Upinder Singh

**Affiliations:** ^1^Division of Infectious Diseases, Department of Internal Medicine, Stanford University School of Medicine, Stanford, CA, United States; ^2^High-Throughput Bioscience Center, Department of Chemical and Systems Biology, Stanford University, Stanford, CA, United States; ^3^Department of Microbiology and Immunology, Stanford University School of Medicine, Stanford, CA, United States

**Keywords:** *Entamoeba*, amebiasis, drug screen, drug repurposing, cyst

## Abstract

Neglected tropical diseases, especially those caused by parasites, are significantly underserved by current drug development efforts, mostly due to the high costs and low economic returns. One method for lowering the costs of drug discovery and development for these diseases is to repurpose drugs developed for other indications. Here, we present the results of a screen of five repurposed drug libraries to identify potential new lead compounds to treat amebiasis, a disease that affects tens of millions of people and causes ~100,000 deaths annually. *E. histolytica*, the causative agent of amebiasis, has two major life cycle stages, the trophozoite and the cyst. The current primary treatment for amebiasis, nitroimidazole compounds, do not eliminate parasites from the colonic lumen, necessitating a multi-drug treatment regimen. We aimed to address this problem by screening against both life stages, with the aim of identifying a single drug that targets both. We successfully identified eleven compounds with activity against both cysts and trophozoites, as well as multiple compounds that killed trophozoites with improved efficacy over existing drugs. Two lead compounds (anisomycin and prodigiosin) were further characterized for activity against metronidazole (MNZ) resistant parasites and mature cysts. Anisomycin and prodigiosin were both able to kill MNZ resistant parasites while prodigiosin and its analog obatoclax were active against mature cysts. This work confirms the feasibility of identifying drugs that target both *Entamoeba* trophozoites and cysts, and is an important step toward developing improved treatment regimens for *Entamoeba* infection.

## Introduction

The protozoan parasite *Entamoeba histolytica* contributes significantly to morbidity and mortality in the developing world and is a leading parasitic cause of death (World Health Organization, 1997). The infectious cycle of *Entamoeba* begins with the ingestion of the cyst, which undergoes excystation in the small intestine to produce the invasive trophozoite form. Trophozoites invade tissue and cause disease symptoms of colitis and liver abscess. Some parasites in the colon convert to the cyst form, which is excreted in the stool and can infect new hosts. Thus, both the cyst and trophozoite forms are important to pathogenesis: cysts transmit infection and trophozoites cause disease symptoms.

All invasive disease with *E. histolytica* should be treated. Additionally in non-endemic countries, the World Health Organization also recommends treatment of asymptomatic colonization with the goal of preventing cyst shedding, as these can be transmitted to household members or close contacts (Stanley, 2003). Treatment for amebiasis is reliant on a single class of agents, the nitroimidazole compounds (i.e., MNZ and tinidazole) (Haque et al., 2003). Advantages of MNZ are that it is highly effective at killing invasive trophozoites, gets to systemic levels to treat liver abscesses, is cheap, and can be orally dosed. However, because MNZ is rapidly absorbed and has poor activity against cysts, it is ineffective in fully treating luminal disease and cysts. Thus, 40% of patients treated with MNZ will continue to have parasites in the colonic lumen (Haque et al., 2003) and a second agent (paramomycin or iodoquinol) must be given to completely clear remaining trophozoites and cysts from the colonic lumen. Other drawbacks to MNZ include unpleasant side effects, alcohol intolerance, and problems with use during pregnancy and lactation (Roe, 1977). The complexity of the treatment regimen increases likelihood of patient non-compliance especially when the second agent is needed at a time when the patient may have clinically improved. Infection with and successful treatment of *Entamoeba* does not confer protective immunity; thus, in countries where the pathogen is endemic, individuals get repeated episodes of invasive disease, and require repeated treatment (Haque et al., 2003). Given that patients are given repeated episodes of treatment and that resistance to MNZ can easily be induced in the lab, it raises concerns that resistant strains may arise (Wassmann et al., 1999).

Given their significant impact on human health, discovery of new therapeutics for parasitic diseases such as amebiasis and other neglected tropical diseases is vital. However, the costs of developing a new drug, which can top one billion dollars (DiMasi et al., 2003), can be prohibitive for diseases mostly found in developing countries. For this reason, screening of repurposed drug libraries, consisting of compounds with known bioactivities and toxicity profiles, has been gaining popularity. Identification of a new indication for a known drug or compound with previously established pre-clinical or clinical data can greatly reduce the costs and time required of bringing a drug to market.

A recent example of this approach in amebiasis is the drug auranofin (Debnath et al., 2012), which recently completed a Phase I trial (Capparelli et al., 2017) to establish safety and pharmacokinetic profiles. Auranofin is a gold-containing compound originally developed to treat rheumatoid arthritis (Minigh, 2007). Screening of a ~700 compound library revealed potent activity of auranofin against *E. histolytica* trophozoites, and *in vivo* efficacy was demonstrated in an animal model of amebic colitis (Debnath et al., 2012). Auranofin was subsequently found to be active against *Giardia* (Tejman-Yarden et al., 2013), suggesting the possibility of its use for a more general treatment of gastrointestinal parasites. Despite the promise of this new potential anti-amebic agent, the path to clinical use is not guaranteed and it is evident that more lead compounds are needed to increase the likelihood of an effective treatment making it to the clinic.

In order to find new drugs targeting *Entamoeba* with a simplified treatment regimen, we aimed to identify compounds that could target both the trophozoite and cyst forms. Because *E. histolytica* cannot be induced to encyst *in vitro*, we used *Entamoeba invadens*, a related *Entamoeba* in which high efficiency encystation can be induced to perform the screen. Both trophozoite and cyst stages of *E. invadens* were screened simultaneously using five libraries, totaling ~3,400 unique compounds; these compounds are enriched for known bioactives and drugs with clinical data, including some FDA-approved compounds. Screening both life stages, we identified three categories of compounds: those that target trophozoites only, those that target cysts only, and those that target both trophozoites and cysts. Following a second round of confirmation in *E. invadens*, select hits were further screened against *E. histolytica* trophozoites. A total of nine compounds had significant activity at ≤10 μM concentration in *E. histolytica. Two* promising lead compounds, anisomycin and prodigiosin, were chosen for further study and characterized for activity against MNZ resistant parasites and mature cysts. This study represents the first successful high throughput screen for compounds targeting multiple life-cycle stages of *Entamoeba*. We have shown that it is possible to identify promising drug candidates that have activity against both trophozoites and cysts and our results give confidence that simplified treatment regimens for *Entamoeba* can be developed.

## Materials and methods

### Parasite culture and strains used

*Entamoeba invadens* (strain IP-1)was grown and maintained at 25°C in LYI media under standard conditions (Ehrenkaufer et al., 2013). For the trophozoite assay, a stable transgenic *E. invadens* cell line expressing luciferase (CK-luc) was established by transfection (Ehrenkaufer and Singh, 2012) and maintained in 40 μg/ml G418 in LYI media. *Entamoeba histolytica* strain HM1:IMSS was grown and maintained at 37°C in TYI media under standard conditions (Diamond et al., 1978).

### Compound libraries

Compounds from Sigma Library of Pharmacologically active compounds (LOPAC 1280: sigmaaldrich.com/life-science/cell-biology/bioactive-small-molecules/lopac1280-navigator), NIH clinical collection (NIHCC: pubchem.ncbi.nlm.nih.gov/source/NIH Clinical Collection), Biomol known bioactives: enzolifesciences.com/BML-2840/iccb-known-bioactives-library, Biomol FDA approved: enzolifesciences.com/BML-2843/screen-well-fda-approved-drug-library and Microsource Spectrum msdiscovery.com/spectrum.html were obtained from the High-throughput Biosciences center at Stanford. A majority of the compounds were maintained in DMSO at a stock concentration of 10 mM. The NIHCC was screened in duplicates while the rest of the libraries were screened in seven-point dose curve with the highest concentration in duplicate for the trophozoite screen. Only the top three concentrations were tested in the cyst screen with the highest concentration in duplicate. Compounds that were active against trophozoites and cysts were re-confirmed in a 384-well format before the secondary assay.

### Compound screening

Compounds (200 nl) were pinned using the SciClone into 384-well plates (EK-30080 white plates for trophozoite screening and EK-30091 clear-bottomed black plates for encystation). CK-luc trophozoites were harvested mid-log phase and seeded with 100 μl media and drug or DMSO control to a final density of 5,000 parasites per well. DMSO only wells were used as a positive control, and wells lacking parasites were used to establish background signal. Plates were sealed to create an anaerobic environment, and allowed to grow for 48 h at 25°C. For the trophozoite killing assay, plates were spun briefly then inverted to pour off media. Bright-GLO luciferase reagent (Promega) was added (20 μl of a 50:50 dilution in PBS) and plates were allowed to incubate 30 min at room temperature to ensure lysis. Luminescence was read on a Tecan Infinite M1000 pro.

For the encystation assay, IP-1 trophozoites were harvested in mid-log phase, washed once in encystation media (47%LG) (Sanchez et al., 1994), and plated at a density of 30,000 parasites per well. DMSO only wells were used as a positive control, and trophozoites were used to establish background signal. Sealed plates were incubated for 48 h at 25°C, then spun and the media removed as with the trophozoite plates. Cysts were stained by addition of 40 μl of 50 μM calcofluor white (Sigma) in PBS, followed by imaging in an ImageXpress micro (Molecular Devices). Number of cysts per well was quantified using MetaXpress software.

### Secondary screen in *E. histolytica* using cell viability assay

*Entamoeba histolytica* trophozoites were seeded in 96-well plates (Corning 3904; black clear bottom) containing 350 μl media and either drug or DMSO at a density of 10,000 cells per well. The plates were then sealed and incubated at 37°C. After 72 h, the media was aspirated and a cell viability dye, fluorescein diacetate (FDA) was added to each well at a concentration of 20 μg/ml, diluted in media. The plates were incubated for 20–30 min at 37°C. The FDA was removed by aspiration and 100 μl of 1X PBS added to each well. Fluorescence was read using the Tecan Infinite M1000 PRO. Effect of the drug was calculated by comparison to DMSO control, after subtraction of background signal. Sources for all compounds are listed in Supplemental Table 1.

### Metronidazole resistant *E. histolytica*

*E. histolytica* resistant to MNZ were generated by continuous growth of *E. histolytica* HM-1:IMSS in MNZ, with steadily increasing concentrations from 1 to 15 μM. Parasites growing stably at 15 μM MNZ were used for further experiments. 20,000 amoebae from the resistant strain were seeded into a 96-well plate with media and 5 μM drug, 20 μM MNZ, or DMSO control and incubated for 72 h. Viability was assayed by FDA fluorescence as detailed above. Two independently generated MNZ resistant lines were used. A wild-type, MNZ sensitive strain was included as a control, with parasite seeding at 10,000 per well as in previous experiments.

### Assay for effect of drug on viability on *E. invadens* mature cysts

CK-luc parasites were induced to encyst by incubation in encystation media (47% LG). After 72 h, cells were harvested, washed once in distilled water, resuspended in water and incubated at 25°C for 4–5 h to lyse trophozoites. Purified cysts were pelleted, counted to ensure equal cyst numbers and resuspended in encystation media at a concentration of 1–5 × 10^5^ cells per ml. One ml suspension per replicate was transferred to glass tubes containing encystation media and drug or DMSO, and incubated at 25°C for 72 h. On the day of the assay, cysts were pelleted and treated once more with distilled water (5 h) to lyse any trophozoites that had emerged during treatment. Purified cysts were then resuspended in 75 μl Cell Lysis buffer (Promega) and sonicated for 2 × 10 s to break the cyst wall. Luciferase assay was performed using the Promega luciferase assay kit according to the manufacturer's instructions. Assays were performed on equal volume of lysate (35 μl) and not normalized to protein content. Effect of the drug was calculated by comparison to DMSO control, after subtraction of background signal.

## Results

### Development and validation of a high-throughput screen against *Entamoeba* trophozoites and cysts

**Figure 1:**
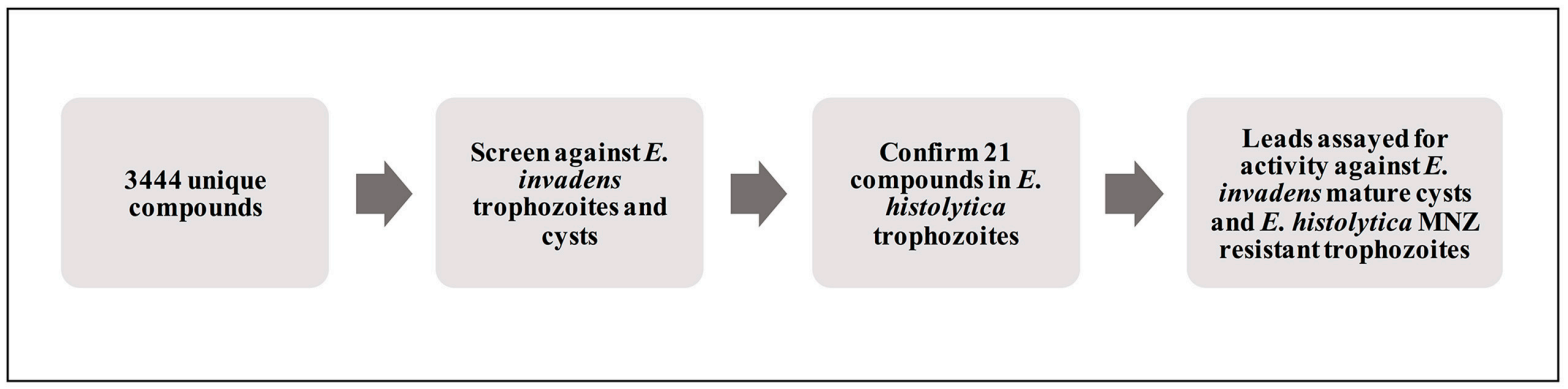
Schematic of screening process. Diagram outlining the process used to identify and characterize lead compounds. Primary screening for activity against *E. invadens* trophozoites and encysting parasites was followed by confirmation of killing of *E. histolytica* trophozoites; select hits were also tested for activity against MNZ resistant *E. histolytica* parasites and mature *E. invadens* cysts.

**Figure 2:**
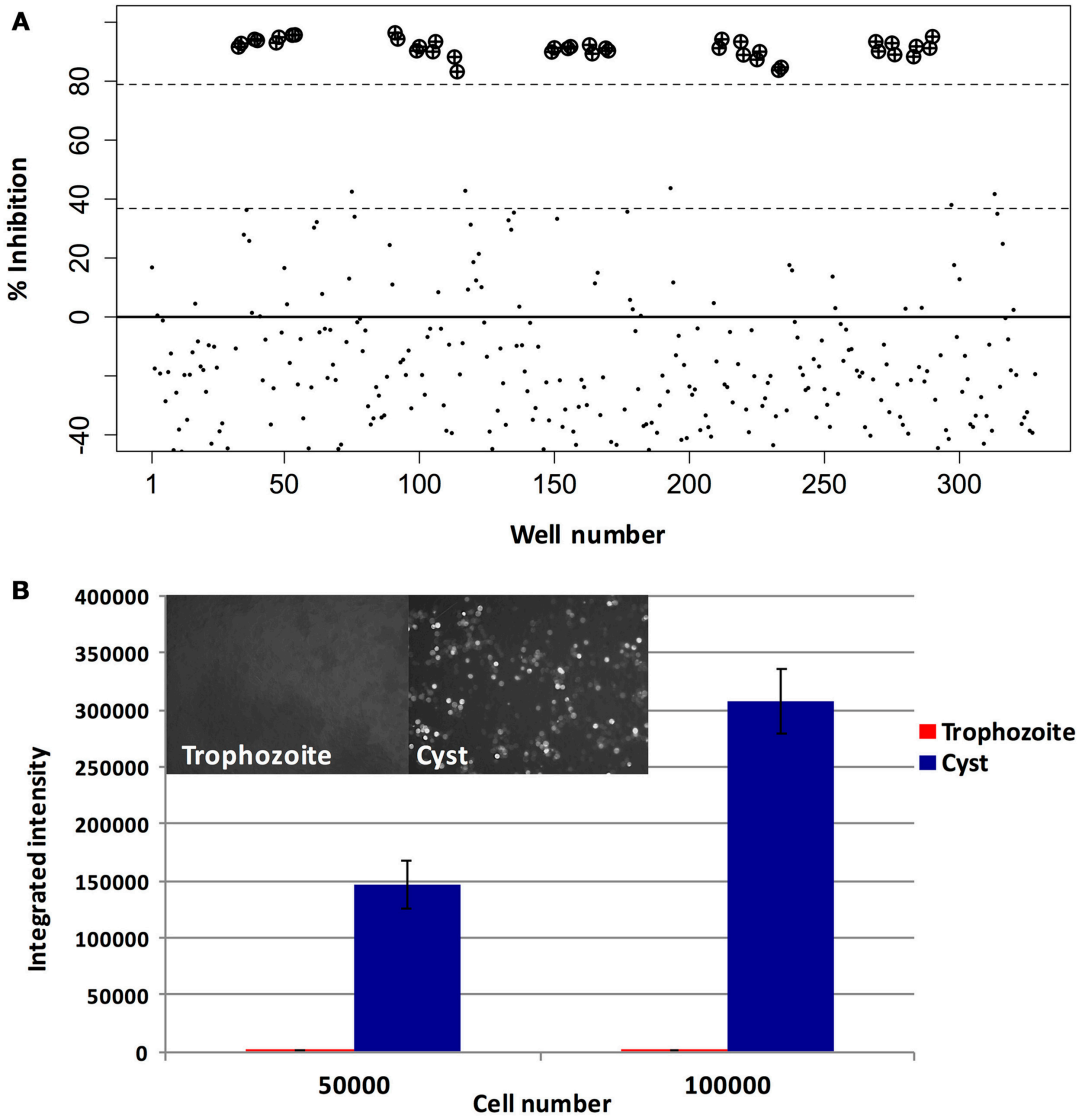
Assay optimization. Validation of trophozoite and cyst growth and drug screening assays. **(A)** Trophozoite growth: *E. invadens* trophozoites were seeded into 384-well plates containing media and either DMSO (0.2%) or MNZ (50 or 100 μM) and assayed for luciferase activity at 48 h. Signal in each well was compared to the plate median and the percent inhibition plotted. Wells containing MNZ are indicated by: ⊕). **(B)** Encystation: *E. invadens* trophozoites were pelleted and resuspended in either LYI or encystation media, then seeded at varying cell densities. After 48 h, plates were spun down, media removed, and calcofluor added to label cyst walls. The plate was imaged and quantified using MetaExpress software. Results of the quantification, expressed as integrated intensity, along with representative images from cyst and trophozoite wells are shown. Background signal coming from the trophozoite wells is very low, and signal in wells with cysts is proportional to the number of parasites seeded.

In order to discover novel drugs with activity against different life stages of *Entamoeba*, we designed a screen to simultaneously test compounds for activity against trophozoites and cysts. As *E. histolytica* cannot undergo encystation *in vitro*, we took advantage of a well-characterized model system for *Entamoeba* stage conversion, the reptile parasite *E. invadens* (Eichinger, 1997). A diagram of the workflow used for screening and identification of lead compounds is shown in Figure 1. To assay trophozoite growth, we used a strain with constitutive expression of luciferase. Parasites were seeded in 384-well plates with drug or vehicle control, sealed with plate sealers to reduce exposure to oxygen. After growth for 48 h, media was removed and luciferase activity was measured. Validation of the assay for well-to-well consistency and ability to detect parasite killing was performed using plates with no drug and plates with spike-in controls of varying concentrations of MNZ. We found significant (>3-fold) reduction in luciferase activity in wells treated with an active compound. Results are shown in Figure 2A; all wells with > 3-fold reduction in luciferase activity, compared to the plate median, were those treated with MNZ (indicated by ⊕). No false positives were noted and little effect was seen from addition of up to 1% DMSO. The Z-factor, calculated using a plate with no drug, was 0.35.

For the encystation assay, mid-log stage trophozoites were harvested and resuspended in encystation media (47% LG), and seeded into 384-well plates containing drug or vehicle control. Parasites were incubated in this media for 48 h to allow cysts to form; the number of cysts was assayed by staining the cyst walls with calcofluor and performing high-throughput imaging to quantify fluorescently labeled cells. Trophozoites, which do not bind calcofluor, were used as a negative control. No signal was seen from wells with trophozoites (image shown in Supplemental Figure 1); wells with cysts showed increasing numbers of cysts based on the number of parasites in the initial seeding (Figure 2B). False positives were minimized by screening plates in duplicate.

Based on these results, we concluded that the assays were sufficiently robust and proceeded with high-throughput screening. Five libraries were screened: NIH clinical collection, Lopac 1280, Biomol FDA, Biomol known bioactives and Microsource spectrum, for a total of 3,444 unique compounds. NIHCC was screened in duplicate at ~10 μM, while all other libraries were screened in a 7-pt dose curve from 20 to 0.31 μM (for the majority of the compounds), with the 20 μM plate duplicated, to allow calculation of an EC_50_. Positive hits were defined as compounds with EC_50_ <30 μM (or >50% inhibition in both plates for NIHCC). Interestingly, we found that some compounds known to kill *Entamoeba* trophozoites, such as the recently identified drug auranofin (Debnath et al., 2012) produced anomalously high calcofluor staining. Visual inspection of phase images captured indicated that cell killing had occurred but with an increase in calcofluor staining, likely due to a stress response leading to chitin upregulation, as has been seen previously in other stress conditions (Field et al., 2000; Aguilar-Díaz et al., 2010) (Supplemental Figure 1). Based on these findings, we considered compounds with low signal (<50% control) as well as compounds with signal >150% over control to be hits for our encystation screen.

After a single round of screening, 167 compounds were found to have activity in at least one assay, with 22 compounds active against both trophozoites and cysts. Significantly, auranofin and MNZ were both present in the Biomol FDA approved library and identified as hits against trophozoites, providing further validation that our screen was able to identify compounds with anti-amebic activity. A total of 63 compounds, including MNZ, were chosen for further testing based on multiple factors including specificity, toxicity, low EC_50_ and activity in both trophozoite and cyst assays; a second round of confirmation screening was performed on these compounds. Of the 63 compounds tested for confirmation, a total of 48 hits were confirmed positive, with the majority (32) being active only in the trophozoite assay. A total of 11 compounds with both trophozoite and cyst activity were identified. Results are in Supplemental Table 2.

### Confirmation of hits in *E. histolytica* trophozoites

As our original and confirmatory screens were performed using *E. invadens*, the next step in identifying potential anti-amebic drugs was to determine efficacy in the human pathogen. *E. histolytica* trophozoites (strain HM-1:IMSS) were grown in sealed 96-well plates with varying levels of drug. After 72 h incubation, cells viability was assayed using the vital dye fluorescein diacetate (FDA). FDA is a cell permeant esterase substrate that is converted by viable cells to yield fluorescein. This probe measures both the enzymatic activity that is required to activate its fluorescence and also the cell-membrane integrity that is required to retain the fluorescence (Medzon and Brady, 1969). We chose FDA as a readout of parasite survival in the *E. histolytica* assay to ensure that the compounds we selected in the luciferase-based assay in *E. invadens* were not selected simply based on luciferase inhibition alone. Percent survival of parasites at 5 and 10 μM drug (compared to DMSO control) are listed in Table 1. Using this assay, we calculated percent survival of parasites treated with MNZ as 96 and 24% at 5 and 10 μM, respectively with a calculated EC_50_ of ~8–9 μM. This is consistent with published reports of MNZ EC_50_, giving us confidence in our assay (Jarrad et al., 2016). Importantly, several drug classes that had promising results in *E. invadens*, including the tetracycline class compounds, showed very poor activity against *E. histolytica*. This discordance between trophozoite susceptibility in *E. invadens* and *E. histolytica* was surprising, and may be due to biological differences between the two species. Of interest, *E. invadens* is usually more robust (for example it needs a higher levels of drug treatment for drug selection with neomycin; Singh et al., 2012), so the finding that some compounds that kill *E. invadens* have no activity against *E. histolytica* was somewhat unexpected. However, robust cell killing was seen with other compounds, including, as expected, the nitroimidazoles (nithiamide, ronidazole, ornidazole). Especially interesting were compounds, such as anisomycin and lycorine, that killed *E. histolytica* trophozoites and were also potent in our screen of *E. invadens* cysts.

**Table 1:** Percent survival of *E. histolytica* treated with select compounds.

**Compound**	**Eh trophozoite % activity at 5 μM**	**Eh trophozoite % activity at 10 μM**	**Ei trophozoite EC_50_**	**Ei cyst %inhibition (mean 20 and 10 μM)**
Prodigiosin	0	0	NT	NT
Auranofin	0	0	3.9	−21.0
Carbadox	18	0	6.5	35.6
Ronidazole	22	0	5.7	49.9
Okadaic Acid	2	1	0.1	Enhancer
Lycorine	46	3	7.6	34.8
Ornidazole	79	7	1.5	45.9
Nithiamide	63	12	8.8	52.0
Aphidicolin	69	39	19.6	88.4
Metronidazole	96	24	24.8	3.4
Conessine	77	63	NT	NT
Emetine	100	63	2.9	−12.9
Chlortetracycline	84	70	3.6	18.0
Thiram	89	75	0.2	0.1
Moxifloxacin	100	75	0.2	47.8
Doxycycline hydrochloride	120	77	0.3	−25.8
Flumequine	99	93	2.1	72.9
Praziquantel	120	110	8.8	−44.6
Xylazine hydrochloride	120	115	7.5	−4.5
Tetracycline	110	120	1.3	−21.7
Levofloxacin	120	125	0.6	−3.3

E. histolytica trophozoites were assayed for viability after 72 h treatment with 5 or 10 μM of each drug using the vital dye FDA. Percent survival compared to DMSO, calculated EC_50_ of E. invadens trophozoites, and percent inhibition (calcofluor signal compared to DMSO controls) of E. invadens cysts (mean of 20 and 10 μM data) are shown. Compounds with high calcofluor staining and confirmed killing by visual inspection are indicated as “enhancer.”

In addition to candidate compounds selected from high-throughput screening, we also examined several compounds which were not in the libraries, but which have historical evidence of use against amebiasis (Balamuth and Brent, 1950; Woolfe, 1963). These include prodigiosin, conessine, and emetine. Of these, prodigiosin, a natural product isolated from the bacterium Serratia marcescens, had very strong activity against *E. histolytica*, (0% FDA signal at 5 μM) and we elected to include this compound in further experiments.

### Target validation of protein phosphatase 2a

Among the most potent compounds with confirmed activity against *E. histolytica*, was okadaic acid, a toxin derived from dinoflagellates, which has been shown to inhibit protein phosphatase 2a (PP2a) in mammalian systems (Xing et al., 2006). While okadaic acid itself is not a promising drug candidate due to its high toxicity and price, a different molecule targeting the same protein could potentially be developed as a therapeutic agent. With this in mind, we attempted to validate PP2a as the target of okadaic acid in *Entamoeba*. First, we searched the *E. histolytica* genome (amoebadb.org) for homologs of PP2a. We identified seven proteins with high similarity (*e*-value < 1e-100) to human PP2a, of which four had all amino acid residues shown to contact bound okadaic acid, including those thought to be responsible for its specificity for inhibition of PP2a vs. PP1 (~100-fold difference in EC_50_). Gene names and alignments with *Homo sapiens* PP2a and PP1 are shown in Supplemental Figure 2.

We then tested the ability of other known PP2a inhibitors, cantharidin, calyculin A, and fostriecin to kill *E. histolytica* parasites (Table 2). Both fostriecin and calyculin A had anti-amebic activity with EC_50_ of 0.001 and 9.4 μM respectively. A third compound, cantharidin, did not kill at concentrations up to 10 μM; this may not be surprising as its activity against human PP2a is the weakest of all the compounds tested. Of particular interest was the activity of fostriecin, which does not inhibit HsPP1 (Swingle et al., 2007) but does have activity against *E. histolytica*. This result may indicate that PP2a, and not other phosphatases, is the target of calyculin, fostriecin and okadaic acid in *Entamoeba*. While further *in vitro* and genetic assays would be required to prove that PP2a is the true target of these compounds in *Entamoeba*, these results are promising and point to PP2a as a potential drug target for *Entamoeba*. PP2a inhibitors, including the compounds we tested, have potent activity against human PP2a, and have been noted for cytotoxic effects against cancer cells; for example, fostriecin has an EC_50_ of ~5μM against HCT-8 cells, a human adenocarcinoma cell line (Jackson et al., 1985). For this reason, further development efforts may be required to identify compounds that selectively inhibit *E. histolytica* PP2a.

**Table 2:** PP2a inhibitors and their activity against *E. histolytica* trophozoites.

**Compound**	**Estimated EC_50_ (μM)**	**IC_50_vs. Hs PP2A (nM)**	**IC_50_vs. Hs PP1 (nM)**
Cantharidin	15	194	1100
Calyculin A	0.001	0.25	0.4
Fostriecin	9.4	3	50000
Okadaic acid	0.02	0.2	25

*E. histolytica trophozoites were assayed for viability after 72 h treatment with varying concentrations (0.001–10 μM) of the phosphatase inhibitors calyculin A, cantharidin, fostriecin or okadaic acid drug using the vital dye FDA and EC_50_s were calculated based on results from 3 independent experiments using the R package drc (Ritz et al., 2015). Potencies of each compound for inhibition of human PP2a and PP1 are included as reference (Swingle et al., 2007). Activity against HsPP2a is a good predictor of anti-amebic activity*.

### EC_50_ calculation of select lead compounds

At this point, we removed two compounds from further experiments: okadaic acid, due to its high toxicity and general unsuitability as a human drug, and ornidazole, which is very similar to the two other nitroimidazole hits. All other compounds with <50% FDA signal at 10 μM concentration (vs. control) were re-tested for *E. histolytica* killing in a dose curve ranging from 10 to 0.01 μM, and EC_50_ and EC_90_ values calculated using the R package drc (Ritz et al., 2015). Results are shown in Table 3. Two non-nitroimidazole compounds, anisomycin and prodigiosin, were found to have EC_50_ values below 1, significantly better than that of MNZ, potentially indicating that a lower dose would be needed for effective treatment. These positive results led us to test two additional compounds in our assay: preussin, an analog of anisomycin (Kasahara et al., 1997), and obatoclax, a synthetic derivative of prodigiosin, which is under development as a drug for lymphoma (Oki et al., 2012). Assaying derivatives of lead compounds can aid drug discovery efforts by identifying compounds with greater efficacy or improved medicinal chemical characteristics. In addition, it can help provide information about the drug target. While preussin had no activity at concentrations up to 10 μM, obatoclax was similar in potency to prodigiosin (Table 3). This result, along with the previous established use in human subjects, where it was well tolerated with mostly transient side effects (Schimmer et al., 2014), indicates the possibility of developing a drug based on the prodigiosin structure as these compounds appear to be highly effective for killing *Entamoeba*, with a potential for a reasonable therapeutic index. Structures for all eight compounds are shown in Supplemental Figure 3.

**Table 3:** Potencies of lead compounds against *E. histolytica* trophozoites.

**Compound**	**EC_50_**	**EC_90_**
Obatoclax (prodigiosin analog)	0.5	1.1
Anisomycin	0.7	1.2
Prodigiosin	0.7	1.1
Ronidazole	0.8	15.0
Carbadox	1.6	14.0
Lycorine	2.5	6.5
Nithiamide	5.0	11.9
Aphidicolin	9.0	64.0

*Selected lead compounds were used to treat E. histolytica trophozoites in a dose curve from 0.01 to 20 μM for 72 h; parasites were assayed for viability using the vital dye FDA. EC_50_ and EC_90_ were calculated based on results from at least 3 independent experiments using the R package drc (Ritz et al., 2015)*.

### Characterization of activity against MNZ resistant parasites and mature cysts

Development of hits from screening into viable drug candidates for neglected diseases depends on multiple factors including *in vitro* efficacy, host toxicity, pharmacokinetic properties, and cost. Using these principles, we chose two main lead compounds: anisomycin and prodigiosin, for further characterization. Anisomycin was of particular interest based on reports of historical use as an anti-amebic agent, including human use (Gonzalez Constandse, 1956; Martin Abreu, 1962), indicating that it likely has an acceptable safety profile. Prodigiosin was chosen due to its potency at low doses and observed rapidity of action. These two compounds were assayed for activity against parasite strains and conditions refractory to current drugs: MNZ resistant trophozoites and mature cysts.

**Figure 3:**
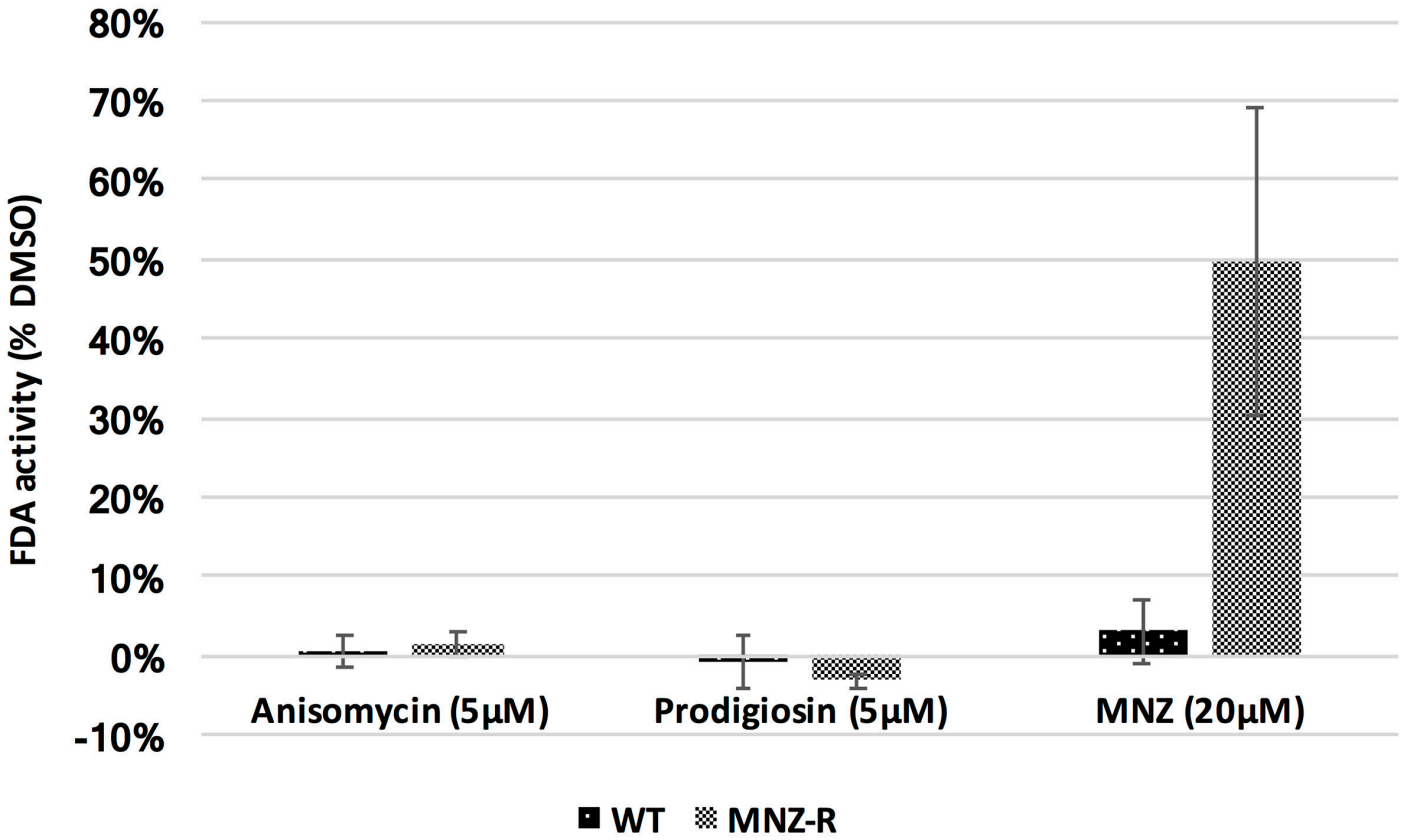
Activity against MNZ resistant *E. histolytica* parasites. *E. histolytica* trophozoites resistant to MNZ at concentrations up to 15 μM, as well as wild-type MNZ sensitive parasites, were exposed to 5 μM of anisomycin or prodigiosin, and assayed for survival after 72 h by FDA fluorescence. Both drugs were equally potent against MNZ resistant parasites (MNZ-R) as against wild type parasites. Fluorescence readings for DMSO, anisomycin and prodigiosin treated parasites is shown. Survival of both strain in 20 μM MNZ is also included. Error bars are ± standard error.

To determine activity of our lead compounds against drug resistant parasites, we grew *E. histolytica* trophozoites in incrementally increasing concentrations of MNZ (from 1 to 15 μM). Once parasites had consistent growth at 15 μM MNZ, they were subjected to our FDA viability assay as previously described. Cellular viability was compared to that of parasites treated with DMSO; a MNZ sensitive line was assayed at the same time and both strains were assayed for MNZ sensitivity to demonstrate that MNZ resistance has been achieved. Anisomycin and prodigiosin both killed MNZ resistant parasites at 5 μM concentrations (Figure 3). The identification of two compounds with activity against MNZ resistant parasites is promising and suggests the possibility that anisomycin or prodigiosin, or related molecules, can be options in cases of clinical MNZ resistance.

Several compounds found in our screen, including anisomycin, strongly inhibited encysting parasites. We hypothesized that some compounds that inhibited early cysts may not be active against mature (48–72 h) cysts, as mature cysts have thick, chitin containing walls, which are impervious to many chemicals (Chatterjee et al., 2009). Therefore, we developed an assay for killing of *E. invadens* mature cysts based on loss of luciferase activity in parasites constitutively expressing luciferase. After allowing the parasites to develop in encystation media for 72 h, cysts were isolated and transferred to fresh encystation media containing drug or DMSO. After 3 days, cysts were treated with water for ~5 h, to lyse any trophozoites that had emerged during drug incubation, and luciferase activity was assayed. Activity was standardized based on number of cysts at beginning of drug treatment, rather than total protein, to account for potential cell lysis during treatment.

**Figure 4:**
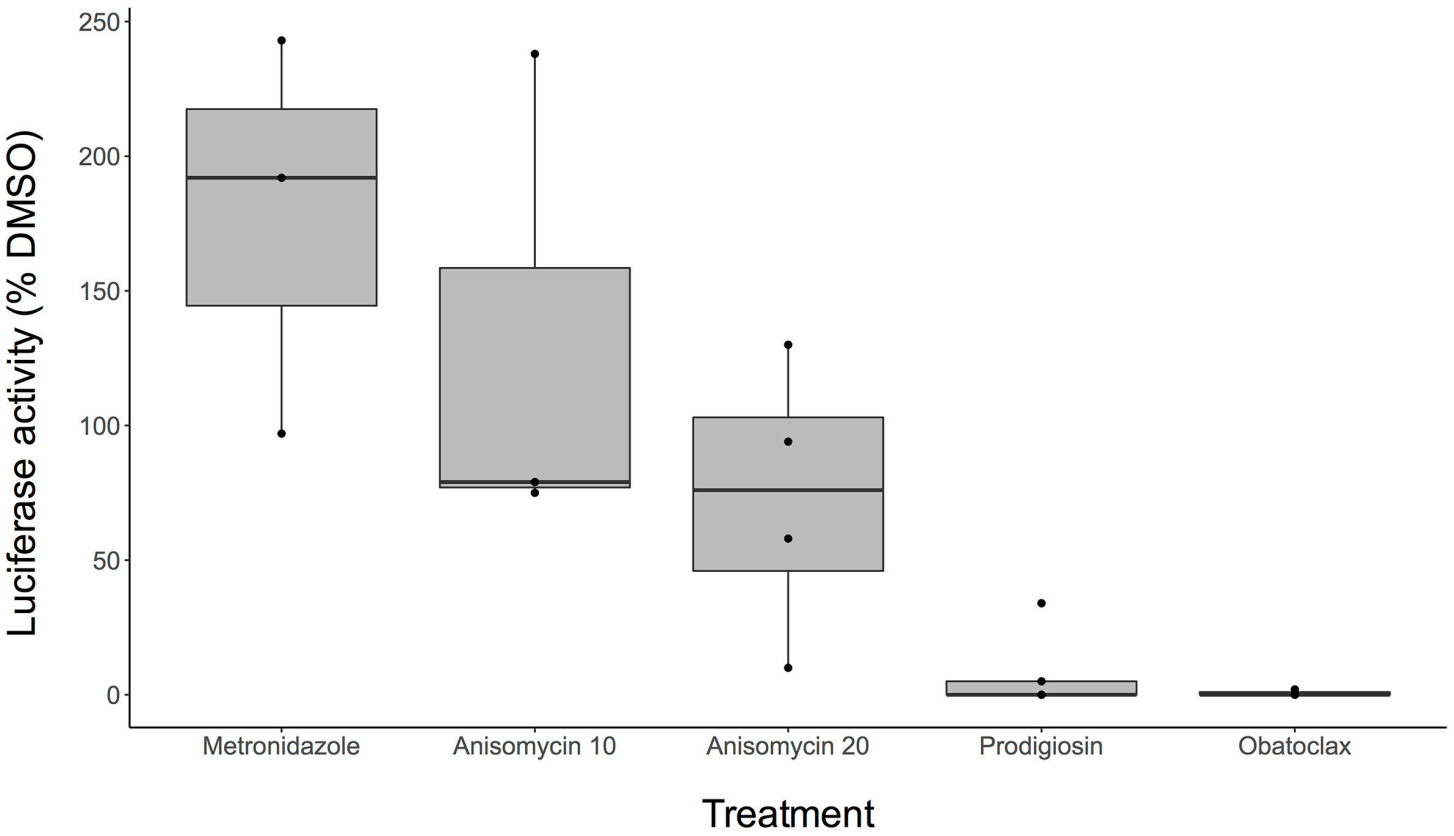
Activity against *E. invadens* mature cysts. Boxplot indicates range of data (percent luciferase activity compared to DMSO control) for at least 3 biological replicates for each drug concentration. Black dots (•) show each separate trial. MNZ was performed at 20 μM concentration, anisomycin at both 10 and 20 μM (labeled as Anisomycin 10 and Anisomycin 20, respectively), and prodigiosin and obatoclax at 10 μM.

Using this assay, we tested the activity of anisomycin, prodigiosin, and its analog obatoclax against mature cysts. We found that both prodigiosin and obatoclax significantly reduced cyst viability at 10 μM concentrations (Figure 4). In contrast, MNZ did not affect mature cysts. Anisomycin, when tested at 10 μM concentration, did not seem to have a substantial effect on mature cysts, despite its inhibition of encysting parasites at this concentration. When tested at 20 μM, a slight reduction in luciferase signal was seen, although this was inconsistent between experiments (Figure 4). Overall, the results suggest that we can identify compounds, such as prodigiosin, that kill mature cysts; however, some drugs with activity against encysting parasites will have reduced potency against fully developed cysts.

## Discussion

The treatment of parasitic diseases is frequently complicated by the fact that many parasites exist in multiple life stages with differing sensitivities to chemical agents. In the case of *E. histolytica*, the dormant cyst form is highly resistant to environmental stresses, as well as to the major drug used to treat amebiasis, MNZ. It would be ideal to have one drug that would potentially treat both forms of the parasite. To this end, we have performed a high throughput screen for anti-amebic compounds directed at both trophozoites and cysts. Using the reptile parasite *E. invadens*, a model for encystation, we identified 11 compounds with activity against both the trophozoite and cyst life cycle stages. After confirmation in *E. histolytica* trophozoites, we obtained five compounds with good activity (EC_50_ <25 μM, >50% inhibition of cysts) against both *E. histolytica* trophozoites and *E. invadens* cysts. Surprisingly, although multiple examples of the tetracycline and quinolone groups of antibiotics were found in the initial screening with *E. invadens*, all of these compounds had poor activity in assays against *E. histolytica*. The two species do have significant differences at the genomic level, including little synteny and variant genome sizes. However, these two classes of antibiotics target biological processes likely to be highly conserved (protein synthesis and DNA topoisomerase). Differences in observed potency therefore may be due to factors such as uptake of the drug by the parasite, or possibly by assay conditions, such as temperature or pH.

One significant aim of a drug screening effort is the identification of proteins that are viable drug targets; this can lead to the discovery of additional molecules that have both the desired activity and have improved toxicity, cost, or other characteristics that make them superior drug candidates. In this screen, we characterized PP2a as a potential drug target in *Entamoeba*, due to the potent parasite killing of okadaic acid as well as several other known PP2a inhibitors. Interestingly, several PP2a inhibitors, including fostriecin and LB-100 (Lê et al., 2004; Hong et al., 2015), have been in Phase I clinical trials due to their potential as cancer treatments. Thus, there is the potential for further development of one of these compounds or other PP2a inhibitors as successful drugs, though delivery and dosage would need to be determined for any use against amebiasis. Alternatively, identification of compounds with greater affinity for EhPP2a than the human enzyme could lead to effective treatments with lower host toxicity.

Aside from the PP2a inhibitors, our screen produced a number of intriguing lead compounds (Table 4), in particular the antibiotic anisomycin, a protein synthesis inhibitor isolated from *Streptomyces*. Anisomycin was ~10-fold more potent than MNZ against *E. histolytica* trophozoites, as well as having activity against *E. invadens* cysts. Further research into this compound revealed previous historical use in humans, for both amebiasis and giardiasis, with good results, although in small cohorts (Gonzalez Constandse, 1956; Martin Abreu, 1962). Side effects were comparable to MNZ: vomiting, nausea, etc. Given the efficacy of anisomycin against MNZ resistant parasites, it could be a useful tool in the event of the emergence of drug resistance in *Entamoeba*. An additional compound, prodigiosin, had very potent activity including against mature cysts. Like anisomycin, there are historical references to prodigiosin as an anti-amebic agent (Balamuth and Brent, 1950); taken together these two cases make an argument for better mining of historical literature for potential anti-parasitic compounds, an approach that has recently proven very fruitful in the treatment of malaria (Tu, 2011). The actual target of prodigiosin in *Entamoeba* is unclear. It has been shown to induce apoptosis in cancer cells, and to alter mitochondrial membrane potential (Montaner et al., 2000; Francisco et al., 2007). While neither of these mechanisms is likely to be active in *Entamoeba*, the target proteins of these pathways may be conserved. Importantly, an analog of prodigiosin (obatoclax), which has been in human clinical trials, had significant activity against both trophozoites and cysts. Development of this molecule as a therapeutic may be possible, given the established safety record in patients.

**Table 4:** Summary of results.

	**_EC50(**E*.*histolytica**)_**	**Cyst activity (*E. invadens*)**	**MNZ resistant parasites (*E. histolytica*)**
Obatoclax	0.5	+++	nt
Anisomycin	0.7	++	Y
Prodigiosin	0.7	+++	Y
Metronidazole	8.9	—	N

*EC_50_ of E. histolytica trophozoites, activity against E. invadens cysts and activity against MNZ resistant parasites are shown. Prodigiosin and obatoclax were considered to have the best activity against cysts, given their ability to kill mature cysts. nt, Not tested*.

In this work we present a high-throughput screen of *Entamoeba* encystation and demonstrate that compounds that are highly active against both *Entamoeba* trophozoites and cysts can be identified. Furthermore, we identified multiple compounds with improved efficacy compared to MNZ, and show that some of the compounds are active against MNZ resistant parasites indicating that they may target an alternate pathway. The work opens up the ability to screen high-value libraries against a neglected parasitic disease and increases the chances that new compounds that are highly efficacious against trophozoites, cysts, and drug resistant strains can be identified. The possibility of using new drugs that affect trophozoites only or cysts only could be enhanced by synergistic effect of the drugs on each. However, further studies are required to determine if using a combination of drugs that affect trophozoite only and cyst only could enhance the potency of the drugs against both forms of the parasite. Future work will focus on further characterization of the lead compounds as development as drug candidates, including lead optimization for toxicological and pharmacokinetic properties.

## Author contributions

GE and SS designed and performed experiments. DS-C provided libraries and helped with data analysis. US conceived of the project and aided in manuscript preparation.

### Conflict of interest statement

The authors declare that the research was conducted in the absence of any commercial or financial relationships that could be construed as a potential conflict of interest.
