# Supplementary material for: High-Throughput Screening of Entamoeba Identifies Compounds Which Target Both Life Cycle Stages and Which Are Effective Against Metronidazole Resistant Parasites
Source: Front Cell Infect Microbiol. 2018 Aug 17;8:276. doi: 10.3389/fcimb.2018.00276 (PMC6107840; doi:10.3389/fcimb.2018.00276)
Supplement: Supplementary file 2 [file Data_Sheet_1.PDF]

Supplemental Figure 1:

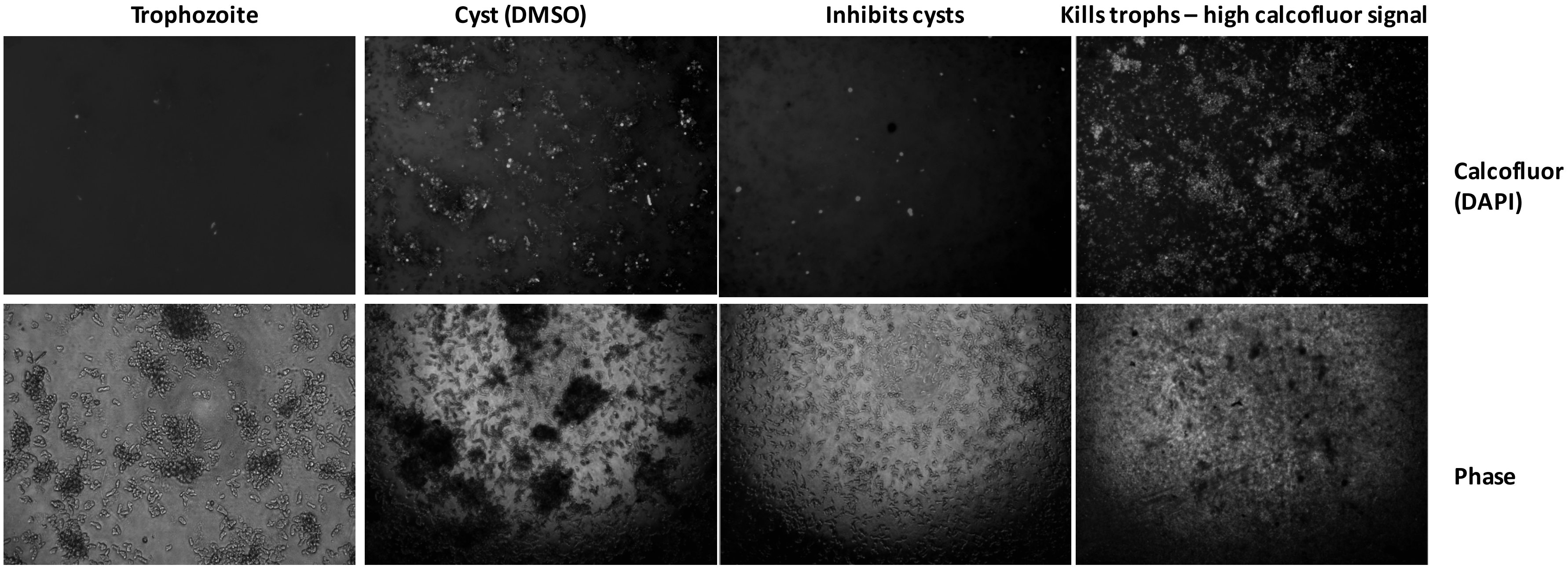

Supplemental Figure 1: Imaging of cells with chitin staining.

Representative images, including both phase and calcofluor fluorescence (DAPI channel) captured during high-throughput screening for trophozoite control and cysts treated with three different types of compounds are shown. Control wells, containing only DMSO, have many labeled cysts and intact parasites can be seen in the phase image. For an inhibiting compound, few cysts can be seen. A third class, which we call "enhancers" give high calcofluor signal after quantitation, but visual inspection of the captured images reveals that there are no structurally intact cysts and under phase few live trophozoites are observed. We speculate that chitin is upregulated in dying parasites as a stress response as previously observed.
