# Supplementary material for: High-Throughput Screening of Entamoeba Identifies Compounds Which Target Both Life Cycle Stages and Which Are Effective Against Metronidazole Resistant Parasites
Source: Front Cell Infect Microbiol. 2018 Aug 17;8:276. doi: 10.3389/fcimb.2018.00276 (PMC6107840; doi:10.3389/fcimb.2018.00276)
Supplement: Supplementary file 3 [file Data_Sheet_2.PDF]

Supplemental Figure 2:

```
Hs_PP1        ICGDIHGQYYDLLRLFEYGGFPPESNYLFLGDYVDRGKQSLETICLLLAYKIKYPENFFL  120
EHI_006050    VCGDIHGQFFDLMKLFSIGGSLPENSYIFLGDYVDRGSYSIEVFTLLMLYKVMYPTRISL  106
EHI_147500    VCGDIHGQFYDLMKLFSIGGSLPENSYIFLGDYVDRGSYSIEVFTLLMLYKVMYPTRIAL  106
EHI_151480    VCGDIHGQFPDFLELFRVGGRVPETNYIFMGDYVDRGINGVEVFMLLIALKVKYPRRVTL  107
EHI_147720    ICGDVHGQYYDLRKLFETGGEVPATKYLFMGDYVDRGYHCLETVLLLLALKVKFPERIIL  107
Hs_PP2A       VCGDVHGQFHDLMELFRIGGKSPDTNYLFMGDYVDRGYYSVETVTLLVALKVRYPERITI  113
              :***:***: *: .**  **  * ..*:*:*****⬆*   :*.. **:  *: :* .. :

Hs_PP1        LRGNHECASINRIYGFYDECKRRYN-IKLWKTFTDCFNCLPIAAIVDEKIFCCHGGLSPD  179
EHI_006050    MRGNHESRQVTQVYGFYEECIKKYGTANSYRWCMEVFDYLNIAALIDGKIFCVHGGLSPE  166
EHI_147500    MRGNHESRQVTQVYGFYEECIKKYGTANSWRWCTEVFDYLNIAALIDGKIFCVHGGLSPE  166
EHI_151480    LRGNHETRQISQTYGFYDECMKKYGNSDVWKMCCDLFDLLALSALIDNRILCVHGGLSPE  167
EHI_147720    LRGNHESRQITQVYGFYDESIRKYKSFNVWKYCTEAFDYLGLAAIIDQKIFCIHGGISPF  167
Hs_PP2A       LRGNHESRQITQVYGFYDECLRKYGNANVWKYFTDLFDYLPLTALVDGQIFCLHGGLSPS  173
              :*****  ⇧:.: ****:*. ::*   . ::   : *: * ::*::* :*:* ***:**

Hs_PP1        LQSMEQIRRI-MRPTDVPDQGLLCDLLWSDPDKDVQGWGENDRGVSFTFGAEVVAKFLHK  238
EHI_006050    IRSIDHIRVIVKRNQEIPHEGAFCDLMWSDPDESVEGMVPSTRGAGYVFGAKPVNEFVQV  226
EHI_147500    IRTIDHIRVVVKRNQEIPHEGAFCDLMWSDPDENVEGMKPSMRGAGYVFGAKPVEEFNHI  226
EHI_151480    IKTLDDISKI-SRFKEAPHDGAFCDLLWSDPEPEVDTWRPSNRGAGYLFGEKATTEFVQS  226
EHI_147720    VNSLDDITKM-ERRQEIPHEGAMCDLLWSDPDPATNDWKSSPRGAGHVFGENNVVKFNHA  226
Hs_PP2A       IDTLDHIRTL-DRLQEVPHEGPMCDLLWSDPDD-RGGWGISPRGAGYTFGQDISETFNHA  231
              : :::.*  :  *  : *⇧:* :***:⬆***:        . ⬆*... ** .    * :

Hs_PP1        HDLDLICRAHQVVEDGYEFFAKRQLVTLFSAPNYCGEFDNAGAMMSVDETLMCSFQILKP  298
EHI_006050    NNIDLIARAHQVVQEGYKYHFDNKLVTVWSAPNYCGRCGNIACIMRVGTDLKQDFTIFDA  286
EHI_147500    NNIDLIARAHQVVQEGYKYHFDNKLVTVWSAPNYCGRCGNVACIMRVGNDLKQDFTIFDA  286
EHI_151480    NKLDFIARAHQIVMEGYDWKFNKLVCTVWSAPNYCYRCGNRASVMELDDLGNQNFLLYDA  286
EHI_147720    NNIDLICRAHQLVNEGYKWCFDKKLVTVWSAPNYCYRCTNDAAIFVIKGENK-QFLTFSA  285
Hs_PP2A       NGLTLVSRAHQLVMEGYNWCHDRNVVTIFSAPNYCYRCGNQAAIMELDDTLKYSFLQFDP  291
              : : ::.****:* :**.:  .. : *::****⬆⬆ ⇧⇧ * ..:: :      .*   .
```

⇧ Unique to PP2a
⬆ Conserved in PP1

Supplemental Figure 2: PP2a alignments

Protein sequences from human PP1 (NCBI: NP_002699) and PP2a (NCBI: AAV38333) were aligned to *E. histolytica* PP2a proteins using Clustal Omega. Residues thought to contact okadaic acid based on the crystal structure are highlighted. Residues conserved in both PP1 and PP2a are marked with a filled arrow, residues unique to PP2a, which may be important for okadaic acid specificity are marked with a white arrow.
