# Supplementary material for: High-Throughput Screening of Entamoeba Identifies Compounds Which Target Both Life Cycle Stages and Which Are Effective Against Metronidazole Resistant Parasites
Source: Front Cell Infect Microbiol. 2018 Aug 17;8:276. doi: 10.3389/fcimb.2018.00276 (PMC6107840; doi:10.3389/fcimb.2018.00276)
Supplement: Supplementary file 4 [file Data_Sheet_3.PDF]

Anisomycin

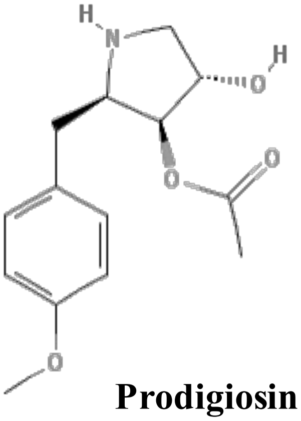

Preussin

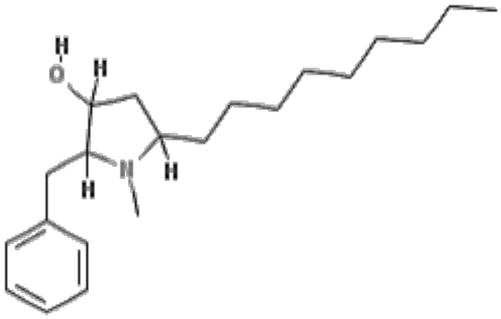

Nithiamide

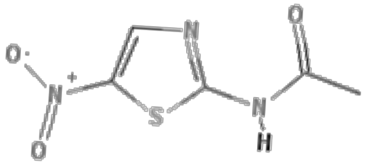

Prodigiosin

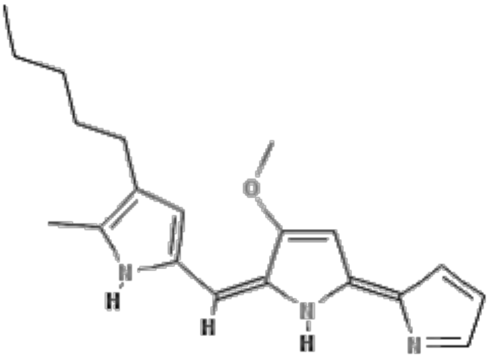

Obatoclax mesylate

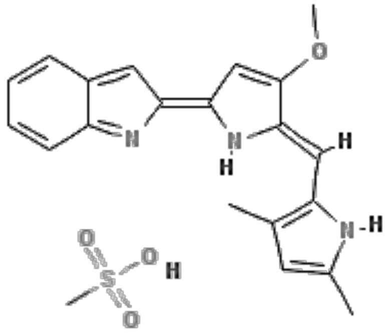

Carbadox

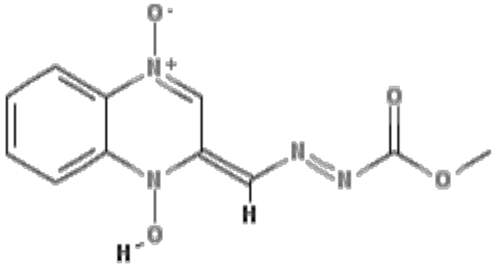

Lycorine

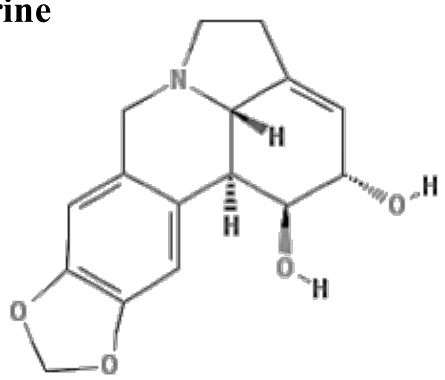

Aphidicolin

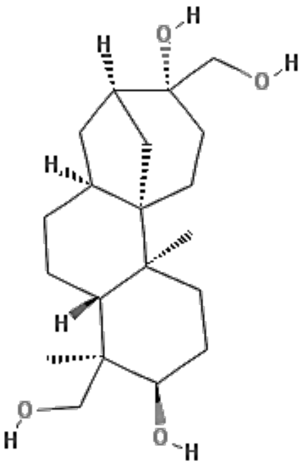

Supplemental Figure 3: Structures of compounds with confirmed activity in *E. histolytica,* as well as tested analogs. All structures downloaded from PubChem.
